# Notices of New Publications

**Published:** 1846-07

**Authors:** 


### NOTICES OF NEW PUBLICATIONS.

*A compendium of Lectures on the theory and practice of Medicine, delivered by Professor Chapman in the University of Pennsylvania; prepared with permission, from Dr. Chapman's manuscripts, and published with his approbation, by* N. D. Benedict, M. D., *&c.* Philadelphia: Lea & Blanchard; pp. 258, 8vo.

This will be a very acceptable volume to the large numbers distributed over the country, who have listenened to the oral teachings of the venerable Professor of theory and practice in the time honored University of Pennsylvania. And for those who have not enjoyed this advantage, it will possess interestfrom the reputation of the author as a teacher and practitioner. It is, in fact, a syllabus of his lectures, excluding those on Eruptive Fevers, Hæmorrhages, Dropsies, Gout, Diseases of the Thoracic and Abdominal Vicera, &c., which had been already published. In glancing over its pages we perceive occasionally adherence to doctrines, which, if decision were to be had of the junior members of the profession, would be declared out of date. This, however, is not surprising. The vigor of intellect which leads to independent original conclusions, is apt to be equally manifested in the pertinacity with which opinions, once formed, are retained. If this be a just criticism, the work is not to be deemed authority on all subjects, nevertheless it is not less valuable as the repository of the results of investigations and reflections continued through a long and highly successful professional career, by one who has occupied and, still occupies one of the most prominent and responsible positions in the medical profession of this country.

---

*Manual of Chemistry, by* Richard D. Hoblyn, A. M., *Oxon.* New-York: Samuel S. and William Wood, 1846: pp. 335, 12mo.

This little work presents a compendium of the fundamental principles and facts of chemical science, intended to serve as an introduction to more elaborate works, and for convenience of reference. The author is favorably known to the profession of this country by his Medical Dictionary, lately republished under the editorship of Isaac Hays, M. D. The present work is well suited to fulfil the objects for which it was prepared.

---

*A Complete Practical Treatise on Venereal Diseases, &c., by* W. M. Acton, *late Externe at the Female Venereal Hospital, Paris, with colored plates.* New-York: J. S. Redfield, 1846, 8vo. pp. 334.

We have already evinced our sense of the value of this work, in devoting a considerable share of two numbers of our first volume to an analysis of its contents, in connection with the treatise of Ricord on inoculation. It is indubitably the best work on venereal affections now before the medical public, and we recommend it to all who desire to possess a text-book embracing the latest and most approved doctrines of the pathology and treatment of this class of diseases. The plates incorporated with the text, will compare very favorably with those accompanying the English edition, which are among the best of the kind we have ever seen.

---

*Clinical Lectures on Surgery, delivered at St. George's Hospital, London, by* Benjamin C. Brodie, Bart. *&c.* Philadelphia, Lea & Blanchard, 1846, pp. 352, 8vo.

As stated by the American editor, in the preface to this work, Sir Benjamin Brodie stands, confessedly, at the head of the Surgical Profession of Great Britain. In their general spirit and practical tone, his lectures bear a strong resemblance to the writings of Sir Astley Cooper, than which, there can be no higher commendation. The lectures which constitute this volume, were originally published in the library department of the Medical News, edited by Isaac Hays M. D.

The interest produced by their publication in that form, has led to their being re-printed in a separate volume. We commend to the careful attention of the medical student, the introductory lecture, which is, in itself, worth more than the price of the whole work.

